# Epstein- Barr Virus: Clinical and Epidemiological Revisits and Genetic Basis of Oncogenesis

**DOI:** 10.2174/1874357901509010007

**Published:** 2015-11-03

**Authors:** Abdelwahid Saeed Ali, Mubarak Al-Shraim, Ahmed Musa Al-Hakami, Ian M Jones

**Affiliations:** 1 Department of Microbiology and Clinical Parasitology, College of Medicine, King Khalid University, Abha 61421, Saudi Arabia; 2 Department of Pathology, College of Medicine, King Khalid University, Abha 61421, Saudi Arabia; 3 Department of Biomedical Sciences, School of Biological Sciences, Faculty of Life Sciences, University of Reading, G37 AMS Wing, UK

**Keywords:** EBV, Epithelial tumors, Latency, Lymphoid tumors, Oncogenes, Oncogenesis

## Abstract

Epstein-Barr virus (EBV) is classified as a member in the order *herpesvirales*, family *herpesviridae*, subfamily *gammaherpesvirinae* and the genus *lymphocytovirus*. The virus is an exclusively human pathogen and thus also termed as human herpesvirus 4 (HHV4). It was the first oncogenic virus recognized and has been incriminated in the causation of tumors of both lymphatic and epithelial nature. It was reported in some previous studies that 95% of the population worldwide are serologically positive to the virus. Clinically, EBV primary infection is almost silent, persisting as a life-long asymptomatic latent infection in B cells although it may be responsible for a transient clinical syndrome called infectious mononucleosis. Following reactivation of the virus from latency due to immunocompromised status, EBV was found to be associated with several tumors. EBV linked to oncogenesis as detected in lymphoid tumors such as Burkitt's lymphoma (BL), Hodgkin's disease (HD), post-transplant lymphoproliferative disorders (PTLD) and T-cell lymphomas (*e.g*. Peripheral T-cell lymphomas; PTCL and Anaplastic large cell lymphomas; ALCL). It is also linked to epithelial tumors such as nasopharyngeal carcinoma (NPC), gastric carcinomas and oral hairy leukoplakia (OHL). *In vitro*, EBV many studies have demonstrated its ability to transform B cells into lymphoblastoid cell lines (LCLs). Despite these malignancies showing different clinical and epidemiological patterns when studied, genetic studies have suggested that these EBV- associated transformations were characterized generally by low level of virus gene expression with only the latent virus proteins (LVPs) upregulated in both tumors and LCLs. In this review, we summarize some clinical and epidemiological features of EBV- associated tumors. We also discuss how EBV latent genes may lead to oncogenesis in the different clinical malignancies

## INTRODUCTION

**Fig. (1):**
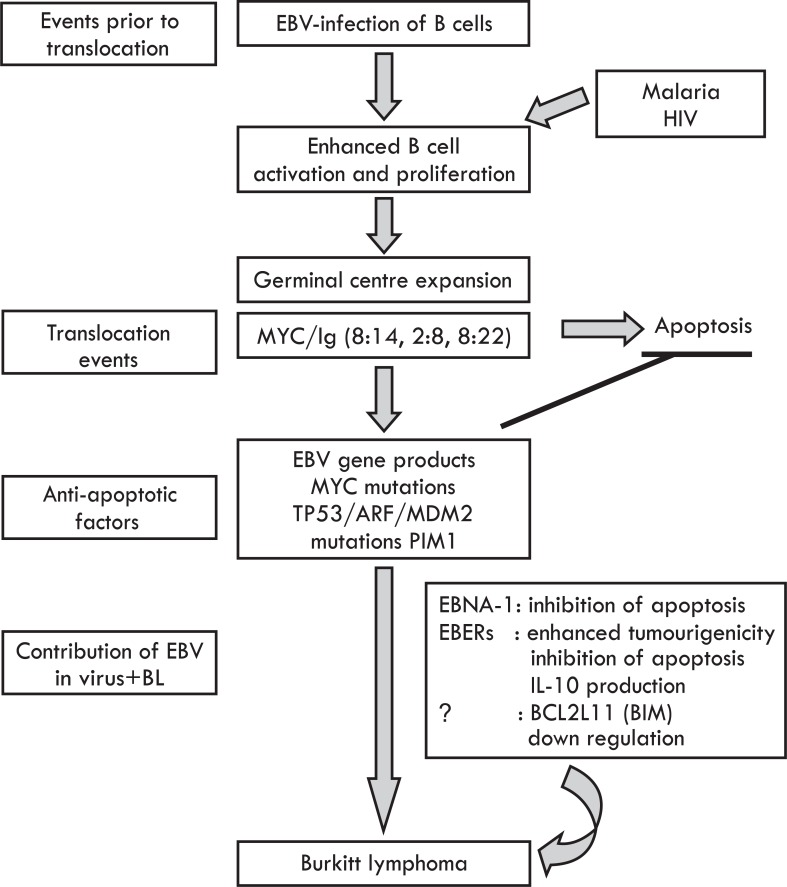
Pathogenesis of Burkitt's lymphoma: This figure indicates the role of some cofactors and EBV genetic entities involved in the development of BL in African population as illustrated by Brady *et al.* (2007) [11].

Epstein-Barr virus (EBV) is classified as a γ-herpes virus [1] and contains a linear DNA molecule about 172 kilo bases in length. It affects more than 90% of adult population worldwide [2]. First exposure to the virus, before or after adolescence, occurs via exchange of saliva or contact with the airborne virus [3]. If the infection does not become clinically silent, infectious mononucleosis is experienced by EBV-exposed persons. This infection was reported worldwide but predominantly in the developing countries and by around 18 months of age, the majority of children in developing countries are found exposed to the virus [4]. Although EBV infection is lifelong, pathogenicity in the host is limitted unless the immune sysytem is compromised. Reactivation of latent EBV results in recurrent infections of a clinical nature. The diseases include those of a lymphocytic nature, namely infectious mononucleosis, Hodgkin’s disease (HD), Burkitt’s lymphoma (BL), post-transplant lymphoproliferative disorders (PTLD) and T-cell lymphomas and those of an epithelial nature such as oral hairy leukoplakia (OHL), nasopharyngeal carcinoma (NPC) and undifferentiated gastric carcinoma [5]. The severity of EBV infection and level of virus titer vary with the carcinoma type with the highest titer for EBV being observed in undifferentiated carcinomas [6]. Infectious mononucleosis (synonyms include Pfeiffer's disease, Filatov's disease, kissing disease, glandular fever or “mono” in North America) [7] is a primary self-limiting clinical syndrome that most frequently affects young adults, although other age groups can also be infected. The classic clinical symptoms of this condition include sore throat, fever, fatigue, malaise, vomiting and loss of appetite with common pathological lesions including lympha-denopathy, spleenomegaly, hepatomegaly and jaundice, all of which may be detected in older adults [8]. Burkitt's lymphoma (or Burkitt's tumor) is a tumor of the lymphatic system (specially B lymphocytes). The name was coined after Denis Parsons Burkitt, who firstly described the disease in 1956 in equatorial Africa [9]. Currently, BL is divided into three main clinical forms: the endemic, the sporadic and the immunodeficiency-associated BL and all forms related to EBV infectivity in African and non-African countries [10, 11] (Fig. **1**) will be described in detail later. Hodgkin lymphoma (HL) is a cancer which originates from the germinal center of B cells with a unique histological picture that comprise inflammatory fields with a minority of neoplastic cells. It differs from the other B cell lymphomas with its characteristic clinical and pathological manifestations [12]. Post-transplant lymphoproliferative disorder (PTLD) is also a kind of B-cell immortalization attributed to immunosuppressive status following chemotherapy for organ transplantation surgery. Such patients can suffer from infectious mononucleosis-like lesions or may develop polymorphic B-cells proliferation which later may become malignant due to additional mutational changes. This disease which is characterized by uncontrolled proliferation of B cells was known to have a strong association with EBV infectivity [13]. 

Although, EBV was originally isolated from Burkitt's lymphoma biopsies, a major B cell lymphoma, it is now considered to have a role in several other epithelioid malignancies [14]. Oral hairy leukoplakia (OHL) is a mucosal tumor firstly recognized and described in 1984. It is a pathological lesion proved to be associated with EBV replication and mostly occurring in people with HIV infection [15]. It was also reported in HIV negative patients when firstly observed in 1996 in a patient suffering from systemic lupus erythromatosis [16]. Nasopharyngeal carcinoma (NPC) is the most common malignant tumor of the nasopharynx, worldwide, also known to be caused by EBV. This carcinoma varies from the others of the head and neck in incidence, causes, clinical pictures and management. It is more common in males than females and in the East Asians and Africans than other racial groups of people [17]. EBV infection was also known as the cause for a reasonable percentage of gastric carcinomas worldwide [18, 19]. The role of EBV in gastric carcinomas was confirmed by detection of the viral gene products like the EBV-encoded small RNA (EBER) in these tumors, in addition to the presence of clonal EBV [20, 21].

Additionally, an *in vitro* EBV infection of resting B cells was known to lead to proliferation, immortalization and consequently to lymphoblastoid cell lines (LCL). These LCL were also shown to be latently infected with EBV, and hence provided a suitable laboratory model for investigation of EBV latency and virus-driven B cells carcinogenesis [22]. LCL have also served as EBV antigen presenting cells in several immunologic methods and tests [23, 24] including the development of human monoclonal antibodies [25, 26]. The efficiency of EBV-mediated CTL proliferation increased with the use of mitogens like phytohemagglutinin and lipopoly-saccharide [27], pokeweed mitogen [28] and some immunosuppressive drugs like the cyclosporine A which prevents the T cell-mediated cytotoxicity of EBV-infected B cells [29-31]. 

In a variety of studies, EBV was proved to perform its oncogenic capacity by an expression of what is known as latent genes, namely the latent membrane proteins (LMP1, LMP2A, and LMP2B) and the EBV-determined nuclear antigens (EBNA1 and EBNA2). LMP1 was reported as the major oncogenic factor of NPC development and were detected in 80%- 90% of NPC tumors [32]. In addition to its direct oncogenic potential, LMP1 was also known to play a role as an immunosuppressive agent against NPC, which allows NPC to develop quietly [33, 34]. The importance of LMP1 as an oncogenic factor was confirmed in several studies by demonstrating that tumor cells are much more sensitive to chemotherapeutic agents when LMP1 expression was inhibited [35]. Previously, the exact contribution of LMP2 and EBNA to cellular tumorgenesis was uncertain but later, due to more advanced research approaches, the role of these factors in the EBV-induced tumorgenesis is well- studied and recognized. Early data suggested that LMP2 was required for tumor cell survival but the more recent data showed that LMP2 plays more diverse and critical functions in the process [36]. LMP2A was proved to downregulate the expression of the transcription factor of NF-κB- resulting in a decrease of LMP1 expression [37]. In addition, LMP2A is responsible for NPC becoming more migratory and invasive [38]. EBNA1 is the factor reported to bind the viral genome to the cellular genome, and in so doing, linking viral DNA replication with the cells division [39]. EBNA2 was known to serve as a powerful LMP1 transactivator [40]. 

Many previous studies have led to the well-established fact that EBV is responsible for tumorgenesis in lymphoid and epithelial cells both during the natural course of infections as well as in the induced lymphoblastoid cell lines (LCL). In this review, we attempt to summarize the clinical outcomes and some epidemiological features associated with the different tumors induced by EBV in both lymphoid and epithelioid cells. We also demonstrate the exact genetic elements involved and the roles played by each separate genetic entity during the transformation process. 

## HISTORICAL BACKGROUND

The history of EBV infection goes back to 1958 when the English surgeon, Denis Burkitt, who was working in Uganda, observed and reported a frequently occurring cancer affecting the children in his work area and equatorial Africa in general [9]. This cancer was later officially named Burkitt's lymphoma (BL) or Burkitt's disease (BD) after the infection was recognized and scientifically well-established. A strong correlation between the distribution of BL and the climatic and geographical conditions in the infection areas was documented; consequently an idea of a vector borne virus responsible for the condition was suggested [41]. Afterwards, using an electron microscopic examination of a biopsy from BL, Epstein, Achong and Barr isolated and identified herpes virus-like particles and hence the virus name in 1964 [42]. As confirmatory evidence, it was shown by serology, in the late 1960s, that BL patients had high antibody titers to the antigens of EBV [43]. Using serological assays, EBV was then also confirmed as the causative virus for infectious mononucleosis and it was also demonstrated as a common etiological agent for undifferentiated NPC development and many other cancers [44, 45]. The transformation capability of EBV was later recognized and documented when the virus was shown to transform resting B cells efficiently *in vitro* and also cause tumors in other primates [46-48]. Subsequently, many research outputs have indicated that EBV is responsible for different human tumors of B cells origin such as Hodgkin's disease (HD) and the lymphoproliferative disorders observed in immunosuppressed individuals. Some T-cell lymphomas and epithelial malignancies were also linked to EBV as will be discussed later. Multiple copies of the circular EBV genome were detected in the tumor cells of all these malignancies and EBV-latent gene expression was confirmed as a major marker in the development of these malignant conditions [49]. In conclusion, to confirm the role of the EBV in the neoplastic transformation of mammalian cells, many research studies pertaining to virology, clinical and the genetics of the virus were intensively carried out by researchers in many parts of the world. They are complied and summarized here for the purposes of this review. 

## GENETICS OF EBV

In total, herpes viruses have been studied intensively at the genetic levels at various points in time [50-52]. EBV, in particular, received a special attention for genetic studies and analysis when recombination techniques became available for the virus in mammalian cells [53-55]. The different genetic components of the EBV genome, and their functions were identified following generation of mutants through cloning techniques. When characterized some of these mutants were also found to have potentially useful medical properties such as those suggested as vaccine candidates [56]. The link between the various genes and biological behaviours such as the transforming property of the EBV was documented in many studies [57-59]. 

The EBV genome consists of double-stranded, linear DNA molecule coated by the capsid protein and surrounded by the tegument layer which is surrounded by an envelope composed of bilayer proteins sandwiched by lipids [60]. The size of the genome varies; it was first reported that EBV genome is about 172 kilo base pairs (Kbp) in length [61] but later it was shown to be about 192 kilo base pairs and to encode about 85 genes [62]. Among all herpes viruses, the EBV genome was the first to be sequenced completely for many strains of the virus [61, 63-65] and recently mapped [66]. A series of repeated DNA sequence accounts for 0.5 kb as terminal repeats at the end of the linear genome observed in the nucleus of cells during latency of the virus in infected cells. They are about 10-20 copies for each infected cell [67]. Long and short sequence domains in the EBV genome were also reported as divided by internal repeat sequences (IRs) and strain variation in the repeat copy numbers was also noted [68].

Based on the sequence organization of the EBNA 2, 3A, 3B, and 3C genes of EBV, two subtypes of the virus have been recognized [69]. The prototype strain of EBV, named as B95-8, was sequenced and the open reading frames (ORFs) and the sites for the transcription processes are readily recognized and the virus has proved to have a coding potential of around 80 characterized and non-characterized proteins [61]. After analysis of the sequences of other EBV strains, more four open reading frames (BVLF1, BDLF3.5, BFR1A and BGLF3.5) have been identified. The function of BVLF1 and BDLF3.5 are unknown so far and BFR1A was suggested to play a role in the DNA packaging whereas BGLF3.5 was recognized as a tegument protein [70]. Generally, various aspects of the EBV genome have been intensively studied to correlate the basic genetic components and their expression with the biological behaviour of the virus, which include infectivity and pathogenesis, immune responses, latency and reactivation and the virus transformation potentia. These studies were especially possible after the advent of molecular approaches in virology. 

## EBV REPLICATION CYCLE

In order to replicate, EBV requires sequential steps of viral protein synthesis. The first expressed proteins are the immediate early proteins which serve as transactivators that enhance the synthesis of the early and late proteins required for DNA replication and production of the progeny infectious virus. The two major immediate early protein transactivators are named Zta and Rta and they function in the initiation of the lytic EBV replicative cycle [36, 71]. The significance of these two transctivators for EBV replication has been well documented through mutants deficient in either Zta or Rta which showed only poor replication levels [72]. Both lymphoid and epithelial cells can be infected by EBV through different attachment and entry mechanisms. To enter B lymphocytes, viral attachment protein (VAP), also called gp350, binds to the CD21 receptor on the cell membrane; additionally, the viral gp42 also attaches to the MHC class II molecules at the cell surface. Both of these interactions allow the viral envelope to fuse to the cell membrane and consequently the virus gains access to B cells [73, 74]. For EBV entry into epithelial cells, viral protein BMRF-2 interacts with the β1 integrins in the cell membrane, and viral proteins gH and gL interact with the cellular αvβ6/8 integrins and these two processes enhance the fusion between the epithelial cell membrane and the viral envelope and ultimately result in the virus entering the cell [75]. Following entry of EBV into the cell, an uncoating process takes place in the cytoplasm in which the virus sheds the protein coat and the viral DNA will be transported to the cell nucleus. In both types of cells, EBV can undergo lytic replication cycle resulting in the production of infectious virions. The lytic replication of EBV in B cells is usually observed to occur following reactivation of latent virus whereas in epithelial cells it has been observed to happen directly in primary infection after viral entry to the cell [60]. The EBV circular latent genome turn to a linear form after reactivation to adopt the lytic replication cycle. Three consecutive stages are required during EBV replication with three types of genes expressed; immediate- early (transactivators), early gene products include BNLF2 serving in replication, metabolism and blockade of antigen processing and the late gene products which express the structural viral proteins like the capsid and the BCRF1 protein, which helps the virus to escape the host immune response. Generally, EBV lytic replicative cycle does not necessarily result into lysis of the cell as the virus is released from the infected cell by budding [60, 76]. Thus while the EBV replicative cycle shows different features at the early phase of replication and entry processes, it adopts similar pathways to complete the cycle in either the lymphatic or epithelial cells.

## EBV INFECTION LIFE CYCLE

EBV is more likely transmitted from infected host to the susceptible host via saliva. Following the primary infection, EBV can replicate extensively in epithelial cells of the nasopharynx and consequently cell lysis releases the virion, and the virus spreads to salivary glands and oropharyngeal lymphoid and epithelial tissues. B lymphocytes are then infected as they circulate in close to oropharyngeal epithelial cells. The virus then circulates throughout the body through the infected B cells. Within the B cells, EBV may either lead to a series of lytic replication cycles or it may show differential expression of the latent genes which will allow the virus to become latent in the cells [77, 78]. As a result of this continuous replication of the virus, viremia will occur and consequently the lymphatic system such as the peripheral B lymphocytes, spleen, liver and lymph nodes will be infected so that EBV can remain latent in these sites as well [49]. When EBV establishes itself as a latent virus it does so as an episomal virus in which the viral genome is found as a self replicating extra chromosomal DNA and is thus known as an episome and latency recognized as episomal latency [79]. Depending on the differential expression of EBV-specific genes, three different types of latencies were recognized, latency I, II and III [49]. For several reasons relating to immunosuppression, EBV sporadically reactivates from latency [77, 78]. Following reactivation, EBV shedding from saliva of infected host will occur intermittently and hence saliva constitutes the major source for transmission from infected to healthy individuals; however, transmission of the virus via blood transfusion, sexual contacts and tissue transplantation have been also reported [80, 81]. Both *in vivo* and *in vitro* infection of EBV leads to transformation and immortalization of B cells [49]. The *in vitro* transformation of B cells can be diagnostically confirmed through the EBV-specific immunofluorescence assay [43] while the *in vivo* transformation resulting in several kinds of malignancies detailed below. Subsequent to B cell immortalization by EBV infection, T suppressor lymphocytes were also observed to play a cytotoxic role and hence the viral counts are reduced with a dramatic reduction in the circulating B cells. 

## EBV LATENCY AND REACTIVATION

As a herpes virus, EBV was known to establish latency after the primary infection in both B cells and epithelial cells where it persists as an episome in the nucleus without productions of virions. The linear DNA molecule of the virus circularizes and only some EBV genes are expressed during latency stage. As mentioned earlier three different latency programs (I, II and III) were reported for EBV; during each program the virus expresses a set of a latency associated genes. The latency programs adopted by the virus in B cells are different from those in epithelial cells and for each type of latency, a distinct set of viral proteins and transcripts will be produced [82, 83]. Within B cells, all three latency programs can take place, starting from Latency III to Latency II to Latency I, whereas upon epithelial cells infection only the latency II program is found. When EBV infects the a B cell, the latency III program starts first with a considerable number of proteins expressed to cause cell transformation. Less gene expression occurs in the move towards latency II to differentiate the B cells into memory cells and finally lesser genes expressed to enter the stage of latency I [84]. The EBNA-1 protein is expressed by the EBV during latency to allow the EBV genome to replicate during memory B cell division. The latency associated with EBV infection of B-lymphocytes is an essential mechanism for virus persistence. The replication of EBV in epithelial cells is necessary for the release of infectious virus into saliva. It was well-established that the EBV Latency III and II infections is a characteristic features of B-cells whereas Latency II is that of the oral epithelial cells and NK- or T-cells all of which can result in different kinds of malignancies [85]. The ultimate and exact requirement of EBNA2 and LMP1 in transformation of B cells *in vitro* studies was confirmed by using of an EBV recombinant deficient in some latent genes. A substantial role for EBNA-LP, EBNA3A and EBNA3C in latency process was also suggested when these recombinants were used [86]. The expression of EBV- latency associated genes during the latency programs is summarized in Table **1**. 

EBV latent infection in B cells can reactivate due to immunosuppression of the infected individual; it can also reactivate *in vitro* to switch to lytic viral replication and this has been accomplished by stimulating the B cell receptor through several mechanisms. It had been observed that patients with polymyositis or rheumatoid arthritis undergo EBV-associated lymphomas more frequently when treated with methotrexate (MTX) than when treated with other immunosuppressive agents. Consequently, MTX was used for an *in vitro* reactivation and release of infectious EBV from latently infected B or epithelial cells [88]. In another study, the potential of monochloramine (NH2Cl), produced during H. pylori infection to the gastric epithelia, to reactivate virus was also examined and the results suggested that NH2Cl induces EBV lytic replication in gastric epithelium if latently infected with EBV [89]. Several *in vitro* studies for EBV reactivation were done with cell lines derived from Burkitt lymphoma as they are very permissive for the lytic cycle of the virus when treated with various immunosuppressive agents as compared to LCLs [90-92].

The issue of understanding EBV latency in infected cells and reactivation from latency as well as the mechanisms adopted for transformation of normal human B or epithelial cells into tumor cells are important for developing and establishing methods to prevent and pave the way for improved clinical management of these EBV-associated neoplasms.

## FACTORS REGULATING EBV-ASSOCIATED ONCOHENESIS

The various factors controlling EBV-associated oncogenesis can be categorized and summarized in the following:

Genetic: The oncogenic capacity and properties of EBV are recognized through its *in vitro* transforming effects. Following infection of primary human B cells *in vitro*, EBV induces proliferation resulting into the development of lymphoblastoid cell lines (LCLs). The genes of six EBV nuclear antigens namely EBNA1, 2 3A, 3B, 3C and EBNA-LP as well as three latent membrane proteins namely LMP1, 2A and 2B are expressed in these induced latently infected cells. Likewise, these proteins are expressed at the early phases of natural infection [93, 94]. EBNA2 is reported as the central transcription factor for both viral and cellular genes expression and is responsible for B cell proliferation and is expressed in LCLs [95]. LMP1 expression, in turn, is regulated by EBNA2 and serves as an active receptor for tumor necrosis factor, an essential apoptotic element [96]. LMP1was also reported to signal in the B cell similar to CD40- CD40 ligand interaction showing similar functional properties with activated CD40 [97]. However, in the lack of EBNA2 in EBV-proliferating B lymphocytes exists, CD40 activation and LMP1 expression leads to similar phenotypic characteristics, the continuous survival of the cell [98]. On the other hand, interestingly both the activated CD40 and LMP1 expression also prevented B cells apoptosis [99, 100]. Additionally, experimentation with a transgenic mice revealed that LMP1 mimics CD40 signaling of B cells differentiation during natural infection [101]. Generally, the role of different EBV-encoded latent genes in tumor formation is recently complied [102] and summarized in Table **2**.

Viral: Strain variation was suggested to be the main factor for the increased incidence of EBV-associated malignancies in specific populations. Some EBV strains indeed possess more distinct biological properties as compared to others. The difference between EBV types 1 and 2 is clearly observed in sequence variation and other EBV variants have been shown based on the viral genome sequence polymorphism of EBNA 2 and 3[103, 104]. Changes among different EBV strains also reside in sequence changes of EBNA1, BZLF1 and LMP1 [105-107]. Investigations into EBV strain variation using restriction fragment length polymorphisms (RFLP) compared EBV strains predominately responsible for NPC in Southern parts of China [108]. Similar EBV genetic polymorphism was also reported when NPC samples from Alaska and Caucasian America were investigated; but no such polymorphism was observed for NPC samples from the Mediterranean and Africa [103]. Genetic analysis for EBV-associated malignancies indicated that the HIV co-infection is another potential factor regulating the incidence of the lymphomas as HIV infection is associated, in most cases, with multiple strains of EBV [109]. 

Environmental: The endemic patterns of many EBV-associated tumors were initially apparent as EBV was originally isolated from samples of African Burkitt’s Lymphoma (BL) [42] and this tumor was observed with high incidence in subequatorial Africa [41]. Endemic cases were also found associated with chronic malaria and EBV infectivity, whereas sporadic cases mostly occurs outside of Africa and were associated with the ileocaecal region, with the jaw less often affected as in the endemic type. Arboviral infection and plant extracted herbs used as therapeutic agents for some diseases have also been suggested as potent tumor-promoting cofactors for EBV induced oncogenesis [110]. The parotid gland tumors and the T-cell lymphomas, due to EBV have been found to develop with increased frequency among Greenland Eskimos and Taiwanese and Japanese respectively [111, 112]. As per the age and gender as factors, patients with T-cell lymphomas were found either middle-aged or elderly, and no gender preference for this disease has been observed [113]. Study of the epidemiology of EBV-associated nasopharyngeal carcinoma (NPC) indicated high incidence occurrence in the southern Chinese and an elevated incidence in Inuit populations and in Mediterranean Africa [114]. The prevalence rates of NPC were particularly elevated in populations of Southeast Asia natives, the Arctic region, North African Arabs and some parts of the Middle East. This was suggested to result from the consumption of preserved food during early ages of life which could predispose to a high risk of NPC development in these populations. Risk factors for NPC development like cigarette smoking, exposure to formaldehyde and wood dust have been also recorded [114]. The co-factors that contribute to the development of Hodgkin’s lymphoma (HL) and gastric carcinomas are currently unknown but environmental and immune components due to immune impairment are likely factors. It is likely that environmental or genetic factors increased infection of distinct susceptible cell populations and activate cellular pathways that are highly synergistic with EBV genes expression which affect cell growth [115].

## EBV- ASSOCIATED LYMPHOID TUMORS

### B-cells Lymphomas

It has been documented in several reports in the medical literature that EBV is frequently linked with many B cells lymphomas, mostly observed in immunosuppressed individuals. Although they look similar on pathological grounds, these EBV-linked B- cells lymphomas were shown to heterogeneous on the molecular and clinical levels, as discussed in the following sections: 

Burkitt's lymphoma: This lymphoma was the first malignancy known to be attributed to EBV. It was firstly observed in central Africa and due to its strict correlation with malaria endemicity, it was suggested that an infectious organism might be involved. Eventually and following EBV recognition, it was confirmed that EBV is the major cause of BL. The natural history of this association was recently reviewed by Moormann *et al.* 2011 [116]. The precise contribution of EBV in the pathogenesis of this lymphoma has been extensively documented. Despite the fact the EBV was detected in biopsies of some BL, some other studies suggested that EBV infectivity occurs before B cells proliferation to induce malignant conversion [117]. EBV is also suggested to have a powerful role in the sporadic form of BL as the episomal defective genome of the virus have been isolated from some sporadic cases of BL tumors in USA [54].

BL has been reported to occur with an incidence rate of about 5- 10 cases per 100,000 children annually in equatorial and central Africa where it became endemic [110]. Higher incidences of this endemic form of BL were also for North Africa, South America and the Middle East regions [118]. Less endemic situations were observed in African countries like Egypt [119] and Algeria [120] where the incidence of malaria is also at a low level. Generally, sporadic BL occurred worldwide but at a lower frequency as compared to the endemic form [110]. Although it can affects adults, BL most commonly affects children where it constitute the majority of B cell lymphomas during childhood, whereas in adults it accounts for less than 10% of all lymphomas [121]. The incidence is reported to peak at the ages of about 5-8 years old and is more commonly observed in males as compared to females [122, 123]. 

At the cellular level, chromosomal translocations within B cells are the consistent genetic feature of all BL tumors. These translocations occur in the region of the c-myc oncogene (mainly involves the 8q24 of the long arm in the chromosome number 8), and in the region of the immunoglobulin heavy chain gene (in the chromosome 14) or in the region of the immunoglobulin light chain genes (in chromosome 2 or 22). The activity of immunoglobulin genes together with the germinal centre of B cell proliferation stimulated through EBV infection were all suggested to be major attributing factors for selection, generation and proliferation of B cells which basically harbor a c-myc translocation [110]. At the virus level, the only protein factor being consistently expressed and detected during BL formation is the EBNA1 protein [35, 124]. In another study, LMP1 together with the EBNA2 were reported expressed in a small numbers of cells in some cases of the endemic form of BL [125]. In an *in vitro* culture of some EBV-positive BL tumors, other EBNAs were also seen expressed beside the LMPs [35].

Clinical presentation of endemic BL mostly includes obvious tumors in the mandible and maxilla with the abdominal organs being involved less frequently. Patients commonly showing swelling of the affected jaw bones and the lymph nodes in the neck, and jaws are rapidly enlarged without tenderness [126]. In the sporadic cases of this lymphoma, abdominal and pelvic organs (namely ileum, caecum and mesentry) are usually involved with the other abdominal and glandular tissues (e.g. tonsils and thyroid) seen affected in some cases but to a lesser extent [127]. Patients of BL are commonly presented to the Gastroenterology clinics with abdominal pain, ascites, abdominal distension and signs of intestinal obstruction. BL has also been observed to occur as a consequence of immunodeficiency, mostly in people with HIV/AIDS infections where it accounts for 30% to 40% of non-Hodgkin lymphoma [128], so much so that it used to be a clinical marker for AIDS. BL was also detected with conditions of congenital immuno-deficiences and organ transplants patients who take immunosuppressive drugs [129].

For accurate diagnosis of BL, lesion biopsy is always performed and CSF and bone marrow are usually examined. Cytology of pleural fluid and ascitis was routinely employed to arrive to a definitive diagnosis. Radiographic applications such as the magnetic resonance imaging (MRI) and CT scanning are applied at diagnosis and then throughout the infection course. Molecular biological techniques were mostly employed to distinguish between BL and the other large B cells lymphomas [130]. Although endemic BL was known to be highly sensitive to chemotherapy, sporadic and immunodeficiency-associated BL are less sensitive [122]. Different regimens of chemotherapy have been tried with different success rates as described by Okebe *et al.* in 2006 [131]. Intensive chemotherapy resulted in a very successful treatment for children with BL. In adults, good results were obtained when chemotherapeutic regimens were used in combination with rituximab (monoclonal antibody) [132]. Non-intensive chemo-therapy with immunosuppressive agents such as cyclophosphamide and methotrexate was proved useful to manage the endemic form of BL in developing countries in Africa as reported by Beogo *et al.* in 2011 [133]. In sporadic or immunodeficiency-related BL, low-intensity infused doxorubicin, cyclophosphamide with vincristine, prednisone and rituximab treatment was seen highly effective, particularly in adults [134]. Bone marrow or stem cell transplants are proposed in recurrent cases of BL and surgery had also been proposed and used if intestinal obstruction is evident and if the abdominal tumors are small and absolutely resectable. The mainstay action for prevention of BL is the vaccine development for EBV and malaria, particularly where malaria is endemic [126]. 

Hodgkin's disease (HD): The first report that suggested HD (also known as Hodgkin's lymphoma, HL) is attributable to an infectious microorganism is made by MacMahon in 1966 [135]. Later, and due to detection of high levels of antibody specific to EBV antigens in the sera of patients with this lymphoma, the infectious agent incriminated as the cause of HD was claimed to be EBV [136]. Furthermore, it was shown that high levels of antibodies to EBV antigens were detected several years before HD development [12]. As a matter of confirmation, several other investigations to confirm the association between HD development and EBV via *in situ* hybridization techniques were carried out and reported [57, 137, 138]. Despite the fact that EBV was detected over the course of HD, cases of EBV-negative HD as a relapse of the previously EBV-positive HD lymphoma have been observed and reported [139]. 

Explicit epidemiological and clinical differences between the EBV-associated and EBV-negative forms of HD tumors has been demonstrated and it has been claimed that HD due to EBV is characterized with multicellularity and commonly observed more in males than females [140]. Another interesting epidemiological feature of HD is that, in USA, EBV-related HD was more commonly observed among Hispanics and Asians than Whites or African Americans populations [33]. Similar findings were observed in studies in the UK where an intimate association between EBV-positive HD and South Asian racial groups of people was noted especially in children [141]. As for age susceptibility, it was observed that EBV-positives cases of HD were more commonly seen in older patients (above 55 years) and younger children (below 10 years) whereas in young adults it is mostly EBV-negative [142]. This fact has led scientists to categorize HD into three disease forms based on the age group of patients: Childhood HD which is EBV+ve (known as MC type), young adults HD which is EBV-ve (known as NS type) and older adults HD which is also EBV+ve (also known as MC type) [142]. The development of HD also correlated with acquired or congenital immunosupprression [39] and the overall incidence of HD was reported to be higher in HIV and/or AIDS patients with most of these lymphomas recognized as EBV-positive [143]. 

As for the genetic basis of this tumor, LMP1 proved to have an essential tumorgenesis of B cells and its high levels of expression was used to explain the obvious clinical and morphological variations between EBV-positive and EBV-negative forms of HD [140]. Some observations had also showed an association between LMP1 and other genes expression suggesting that LMP1 upregulated these genes in an *in vitro* primary HRS cell cultures (e.g. IL-10 and TRAF1). Therefore, in contrast to EBV-negative HD forms, these genes are more likely to be expressed in EBV- positive HD [144-146]. Immunohistochemical assays and genetic studies using biopsies of HD also revealed that cultures of HRS cells of EBV-positive HD cases express LMP1 to high levels but not EBNA2 [147-149]. 

The most salient symptomatology of HD is the painless swelling in one or more lymph nodes especially those of the neck, underarm and the groin. Some other patients have symptoms like fever, night sweats, fatigue, total body itching, weight loss, bone pain and increased susceptibility to infections as recently reviewed by Movva [150].

Detection of abnormal cells, referred to as Reed-Sternberg cells, by the pathologist following biopsy of an enlarged lymph node is the first line of HD diagnosis. Radiographic imaging such as CT scanning, MRI, or PET scanning may also be employed to determine the stage of HD. Immunohistochemistry was also employed as the constitutively nuclear encoded NF-κB from the HRS cells can be detected [151]. The treatment of Hodgkin lymphoma (HL) depends on the direct medical intervention using chemotherapy and radiotherapy; stem cells transplantations were also recommended in relapsed cases, although about 20% of patients were seen not responsive to that treatment [152]. Brentuximab vedotin, which was approved in 2011, made great shifts in the treatment of HL and other novel therapeutics are expected to pave the way for future improved HD treatment [153]. A combination of Brentuximab vedotin and Rituximab was also implemented to help the immune system kill lymphoma cells with some side effects encountered [154]. Radiation therapy to kill lymphoma cells had also been tried to shrink tumors and help control pain with better results obtained when combined with chemotherapy [155]. 

Post-transplant lymphoproliferative disorders (PTLDs): They are defined on pathological grounds as combinations of lesions progressing from atypical polyclonal B cells proliferation in immunodeficient individuals to more aggressive monomorphic lymphomas which continue as malignancy even after immunity reconstitution [156]. On clinical grounds, they are defined as lymphoproliferative disorders observed in the tissue transplanted patients as they are usually under immunosuppressive therapy and also in those who are congenitally immunodeficeint individuals such as those suffering from Wiscott-Aldrich syndrome and X-linked lymphoproliferative syndrome. They are also frequently observed in AIDS patients who may also experience similar tumors of B-cell origin [157]. Although many of these B cell proliferation conditions derived from the patient himself, in some cases these proliferated B cells were reported originated from the donor [158]. The overwhelming majority of these PTLDs were confirmed as associated with EBV infectivity and most of them characterized by the latency III pattern of expressed genes, although Latency I and Latency II forms of EBV were occasionally also observed [159]. In these PTLDs, EBV-negative cases were also reported; however, they are more seen as T cell lymphomas than B cell tumors and characterized by cellular monomorphic picture, later presentation and more aggressive as compared to the EBV-positive tumors [160, 161]. 

The variations among the PTLDs in their incidences and clinical manifestations were seen as dependent on the number of the immunosuppressive drug used and their doses, the period of the immunosuppressive status and the type of the tissues transplanted. Common clinical presentations are frequently observed as diversified extranodal lesions such as those seen in the gastrointestinal tract or in the organ transplanted. The frequent occurrence of these PTLDs in the organ transplanted was explained by the fact that continuous and chronic antigenic stimulation in the graft tissues stimulates the development and pathogenesis of these lesions. In another study involving SCID mice, it was reported that T cells were also mandatory for PTLD like tumors to develop and this suggests a crucial role of T cells in the growth of these B cells lymphoid tumors [162].

At the cellular level, chromosomal imbalances and genomic alterations in EBV positive PTLDs were detected in some studies and were also proposed to play an important role in the development and progression of EBV induced-lymphoproliferations and their clinical significance [163, 164]. On the EBV side, LMP1 is the major proto-oncogene that is expressed in most EBV-positive PTLDs and there is an intimate association of EBV LMP1 deletion mutants with the PTLDs morphology, PTLDs clonality and EBV strain subtype [165].

Antiviral chemotherapy, cytokine therapy, cytotoxic chemotherapy, anti- B cells antibodies, cellular immunotherapy and local treatments have all been observed to successfully cure these conditions [166].

### T-cells Lymphomas

Beside B cell lymphomas, EBV was also confirmed in several studies to be associated with multiple kinds of T-cell lymphoma. The EBV genome was detected in some T cell lymphomas in the sinuses and nasal cavity in patients from many parts of the world e.g. Peru [167], Japan [168], USA [169], Taiwan [170], some European countries [171] and recently from China [172]. In other studies, EBV was detected to account for about 50% of T-cells lymphomas which indicates that the tumor establishment may precede EBV infection [173]. That was confirmed following the advent of more sensitive molecular biological techniques which investigated the role of the EBV in the development of these T cell lymphomas. The association of the EBV infectivity with the T-cells lymphomas was also reported to be site-specific i.e. EBV was detected in 100% and 20% of the nasal and gastrointestinal lymphomas respectively and rarely in primary cutaneous T-cell lymphomas [174]. 

Peculiar phenotypic and genotypic manifestations for the sinonasal T-cells lymphomas were observed; these include the expression of some cellular markers, absence of T cell antigens and receptor gene rearrangements. However, the latter was detected recently in some cases of skin T cells lymphomas [175]. It was also reported that most of the EBV-positive T cell lymphomas are extranodal and have a cytotoxic phenotypic features [176]. This suggests that such kinds of lymphomas may occur as a result of EBV infection of CTLs during the killing action of EBV-infected cells. As a matter of etiology and clinical outcome, the nasal NK and T cell lymphomas are considered the most closely associated with EBV as well as the most aggressive subtype of non-Hodgkin lymphoma (NHL) ever recorded. 

In a genetic study, the mRNAs of EBNA1, LMP1, LMP2 or BamHI A transcripts in the EBV-positive peripheral T cell lymphomas were detected suggesting their expression. This study also showed and indicated that EBV-positive T cell lymphomas were clonally expanded from a single EBV-infected cell with a gene expression pattern different from that observed in Burkitt's lymphomas or PTLDs but seen similar to that of the NPC [177]. 

As mentioned earlier, despite the fact that EBV-related post-transplant lymphomas (PTLPDs) are predominantly B-cell lymphomas, a reasonable number of them are known to be T cells-associated. These T-cell PTLPDs were observed to be inconsistently associated with EBV infectivity [178]. Based on the site of their development, multiple clinical and epidemiological presentations were reported for EBV-associated T cell lymphomas:

Peripheral T cell lymphomas (PTCL): They are diversified types of T cells lymphoproliferative diseases characterized by different pathological and clinical manifestations. As compared to B cell lymphomas, PTCL are rare but more difficult to manage and treat. PTCL are the most frequently occurring among all types of T cell lymphomas. In North America, PTCL accounts for 10–15% of all non-Hodgkin's lymphomas in and known to involve mature T cells or NK cells [179]. Although most of PTCLs were diagnosed and recognized confined to the lymph nodes, other tissues such as GIT, liver, skin and bone marrow were also seen involved; such kinds of PTCLs are more aggressive and cannot resolve without combined chemotherapy when diagnosed. A particular subtype of PTCL known as the peripheral T-cell lymphomas non-otherwise specified (PTCL- NOS) is the most commonly recognized subtype. PTCL-NOS usually showing extranodal lesions in the liver, GIT, spleen and bone marrow [180]. The role of EBV in causation of these kinds of lymphomas has been documented [181, 182].

Anaplastic large cell lymphomas (ALTCL): They are group of lymphomas characterized by proliferation of the large lymphocytes and peculiar growth characteristics and expression of the cytokine receptor CD30. They were firstly described in 1985 as different types of T cell lymphomas and known to account for 12- 15% of all T-cell lymphomas in adults and around 10- 30% of all lymphomas in children. Depending on the site where they develop, ALTCL were categorized into three types: systemic lymphomas, which are usually present in lymph nodes, namely the anaplastic lymphoma kinase (ALK) positive and ALK negative lymphoma, based on the presence of the ALK protein on the surface of the malignant cell and a third non-systemic type, appearing only on the skin, which is also called a primary cutaneous anaplastic large cell lymphoma. As compared to the skin type, the systemic lymphomas are mostly fast growing [183]. Although the classification of the WHO for the lymphoid and hematopoietic tumors, 2008 edition, stated that ALCL is consistently negative for EBV, some studies have reported that a correlation between EBV and ALCL does exist [184, 185]. A case of ALCL in immunocompetent 35-year-old male using an *in situ* hybridization was also proved positive for EBER in the large neoplastic cells [186]. 

Angioimmunoblastic Lymphoma (AIL): EBV had also been proposed as a possible causative agent involved in the pathogenesis of AILS which is a progressive and often fatal lymphoproliferative disorder. It is a fast-growing T-cell lymphoma with a primary symptomatology including swelling of the lymph nodes as well as systemic symptoms like fever and rash. Generally, this lymphoma is treated as for the other fast growing systemic lymphomas although it may only require mild therapy in certain conditions [187]. Previous findings also suggested that in this type of lymphoma the T cells are very vulnerable to EBV persistent infection and that it is more likely to give a growth advantage to infected cells. It was concluded that the pathogenesis of these AIL-TCLs is mediated by three factors, EBV infectivity, cellular genetic defects and impairment in the immune system [188].

## EBV- ASSOCIATED EPITHELIAL TUMORS

In addition to its role in the hematologic malignancies, EBV has also been known to be implicated in both epithelial and mesenchymal neoplasms. The mechanism whereby EBV enters epithelial cells, previously cited in this review, was debated for some time, and some studies have confirmed a role for CD21 as receptors for the virus in human epithelial cells [189, 190]. These receptors seen to play a similar role in the fresh tissues of the liver, skin, GIT, endothelium, renal tubules, striated muscles and parotid glands [191]. It was also reported that the secretory component-mediated IgA transport, in which the mucosal polymeric immunoglobulin A (pIgA) binds to a secretory component, a transmembrane protein expressed by the epithelium; and is then endocytosed by the cell, was also proposed as a potential means for EBV infection of endothelial cells [192]. The mechanism of cell to cell contact between virus releasing cells and non-infected epithelial cells for EBV entering and infection was also reported [193]. 

Nasopharyngeal carcinoma (NPC): It is the first epithelial carcinoma known to be associated with EBV infectivity [194, 195]. This was confirmed by the elevated antibody titers against EBV in many cases of NPCs [196] and later when EBV DNA was identified in extracts of NPC [197]. The EBV genome was also detected in NPC tissues using Southern blot technique suggesting that EBV infection precedes the clonally expanded malignant cells of NPC [198]. It was well established that three factors are involved in the NPC incidence, EBV infectivity, genetic predisposition and some environmental factors which may vary from one population group to another [199].

Undifferentiated type of NPC (UNPC) was commonly observed in Arctic, Middle East (mainly North Africa), China and South-East Asia. It was also observed that people of Chinese descent are the most common population showing higher incidences of NPCs. Beside these genetic and racial predisposition, an environmental factors such as food components (e.g. salted fish) are known to be important contributing factors in NPC development [200]. Other factors including history of respiratory infections, tobacco smoking and consumption of preserved foods were also considered environmental factors for NPC development [201, 202]. As for gender as a co-factor, it was recognized and reported that NPC incidences are 2 to 3 fold higher in males than females in the populations investigated [203]. Based on histological bases, WHO classified NPC into three types: type I (keratinizing squamous cell carcinoma); type II (differentiated non-keratinizing carcinoma) and type III (undifferentiated NPC) [204].

Looking at the genetic basis of NPC development, it was confirmed in some studies that individuals who inherit certain human leukocyte antigen (HLA) gene alleles, that have reduced potential to present EBV antigen to immune cells, were proved to have an increased risk to develop NPC, whereas individuals with HLA alleles that present EBV efficiently may have a lower risk for NPC [205, 206]. On the EBV side, LMP1 and LMP2 were proved to have a strong influence on the cells genetic expression and proliferation leading to highly invasive and malignant growth of NPC. Additionally, the establishment of EBV latency and transformation in epithelial cells is considered as a major contributing element in the development of NPC [207]. In another former study, using selected human sera and immunoblotting, it was revealed that EBNA2, EBNA3 and EBNA-LP were not expressed in NPC tumor cells [208]. However, the EBNA1 and EBERs proteins were observed to be expressed in all EBV-positive NPCs [208, 209]. Expression of LMP2A gene in NPC tumors was also detected using PCR while the LMP2A protein was not detected in NPC tumors [210].

Clinically, patients with NPC may be presented with one or more of the following symptoms; nasal obstruction involving nasal discharge, congestion and bleeding. Hearing changes which are usually attributed to the blockage of the Eustachian tube may also be seen. Palsies of the cranial nerve due to the extension of the NPC tumor into the skull base causing headache and neck swelling were also observed in some cases as described by Sham *et al.* (1990) [211]. Some other malignancies with similar clinical features to NPC in sites like tonsils, lungs, skin, thymus, salivary glands, stomach, breast and uterine cervix were also observed and referred to as undifferentiated type of NPCs [212-216]. 

Gastric carcinoma (GC): Diffuse gastric or intestinal adenocarcinomas were detected and also proved to be associated with EBV in about 10% of the total cases throughout the world [20, 217, 218]. EBV-negative GCs were also reported in the majority of cases [219]. 

EBV-positive GCs were more commonly observed to occur in males than females [20, 218]. No age preference for the occurrence of these kinds of carcinomas was observed, however, some studies' findings revealed more EBV-positive tumors in older people compared to children or young adults [38, 219, 220]. They were more likely to occur in the cardia or body of the stomach than the gastric antrum [6, 218, 221] and most of them were detected as undifferentiated tumors [6, 38, 218, 222]. EBV-positive GC is a non endemic disease detected in all parts of the world. However, high incidence rates of the disease were recognized in certain regions with the highest incidences recorded in Germany and USA and the lowest in China [20, 223-226]. In an epidemiological study in USA, EBV-positive GC was more commonly observed among Hispanics than in Whites or African Americans. This indicates that the correlation of GC development with the EBV infectivity varies with the different ethnic groups of population [227].

Advanced molecular studies for these type of carcinomas showed a restricted genetic expression which was confined to EBERs, EBNA1 and the BZLF1 with a reasonable expression of either LMP1 or the other EBNAs [21, 228]. Also, EBV- encoded small RNAs were seen expressed in every GC cells, this indicates the importance of this molecule in development and persistence of the GC [229]. In another proteomic study, EBV-positive GC was found to have distinct protein expression profile and clinicopathological features as compared to EBV-negative carcinoma [230].

As for the clinical presentation of EBV-associated GCs, they appear as tumors in the non antrum part of the stomach accompanied by ulcers in the upper part of the stomach when endoscopy was used. It is unlike chronic gastritis and carcinoma attributed to Helicobacter pylori (Hp), which locates predominantly in the antrum of the stomach [229]. However, Yani *et al.* in 1999 [231] showed that EBV-positive GCs are commonly observed adjacent to the mucosal atrophic border, where mild to moderately chronic atrophic gastritis (CAG) is commonly observed. Also EBV and Hp were frequently detected in the stomach mucosa with moderate CAG, the area where the inflammatory cells are in common and not the mucosa with obvious CAG, where inflammatory cell infiltration is minimal.

Oral hairy leucoplakia (OHL): It is also known as HIV-associated hairy leukoplakia [232] and defined as white patches on either sides of the tongue with hairy appearance which cannot be scraped off. This carcinoma was firstly reported and intensively described by in 1984 Greenspan *et al.* [233]. As a confirmation for the association of this malignancy with EBV, an electron microscopic examination revealed a herpes virus-like particle. Later, the role of EBV in this tumor was confirmed using the DNA hybridization and immunohistochemical techniques [234]. A cyclovir treatment of this tumor, an inhibitor of the herpesvirus DNA polymerase, resulting in the regression of the OHL and hence EBV was implicated as a causative agent in its development [234]. This condition was confirmed to be due to EBV infectivity when the virus reactivated from latency because of immunosuppression [235]. OHL was frequently observed as common oral lesions with HIV/AIDS and oral candidiasis [236]. This tumor was reported as the most commonly HIV/AIDS associated condition and was known mainly to occur in adults rather than children and more in males than females [237]. Firstly, OHL was believed is the disease of HIV-infected and homosexual males but later was found to occur in people other than these two groups [237].

To determine the genetic behavior of EBV during OHL oncogenesis, a genomic study using Northern blotting and sequencing to study the EBV gene expression dealing with clones from a cDNA library constructed using RNA isolated from OHL biopsies was made. This analysis revealed the expression of BdRF1, BCRF1, gp350/220, BARF0, and BKRF4 reading frames whereas EBNA1, EBNA2 and EBNA -3A RNA were not seen expressed [238].

No exact symptomatology and epidemiological patterns connected to this tumor were reported. However, generally symptoms and signs linked to immunosuppression were the majors symptoms to be detected. Lesions were described previously as white patches on the lateral surfaces of the tongue and may involve the dorsum of the tongue but they are rarely seen on the buccal mucosa, pharynx, esophagus and soft palate. These lesions textured as vertical corrugation (hairy), or as thick furrow or appears shaggy [239]. 

## EBV STRATIGIES FOR IMMUNE EVASION

EBV immune evasion is the principal issue to be highlighted here as the well-known lifelong persistence of the virus in the infected cells which has been attributed to the virus' strong potential to escape from the host's immune responses. EBV was recognized as a powerful immunogenic virus upon infection. This was clearly demonstrated by the high levels of the immune responses elicited, especially during the primary infection. However, in order for the EBV to survive and successfully establish itself as a latent virus in the memory B cells of the adult, it has a farrago of mechanisms to escape the host immune responses. These include shut down of the most immune responsive proteins via the lytic proteins which interfere with antigen processing and with expression of the MHC molecules in the infected cells. Additionally, viral homologues of human cytokines are produced by the infected cells to mimic host antigens. These strategies and mechanisms for EBV-mediated immune evasion were intensively reviewed by Ressing *et al.* [240]. The limited subset of viral gene products during latency as compared to the expression of about 80 EBV gene products detected during the replicative phase is also considered a kind of immune evasion [240]. To specifically address this issue, a protein encoded by EBV, namely BNLF2a, had been identified and normally expressed in the early phases of the lytic replication cycle of the virus. This protein was known as an immune evasion protein. It was recognized to inhibit the peptide- and ATP-binding functions of the transporter associated with the antigen processing steps during the immune response. In addition, this protein expression resulted in decreased MHC class I expression and thus prevented the presentations of the viral antigens to CD8+ CTLs [241] and EBNA1, a major latent gene products is a poorly recognized protein by CD8+ T lymphocytes [240]. It was also recently shown that the BCRF1 genes encoded by the EBV, which are homologues of IL-10, are immunoevasins in EBV's lytic cycle [242]. 

Despite all these strategies adopted by EBV for immune escape, the development of the previously mentioned tumors has been attributed to some imbalances in the equilibrium between the inherent virus transforming properties and the host immune system [243]. Overall, the evasion by EBV of the host's immune responses allows the virus to remain in the body as a latent infection for life whereas reactivation of the latent EBV comes as a consequence of immunosuppression and the reactivated virus may transform the infected cells. 

## CONCLUSION

The present review provides comprehensive coverage of some epidemiological and clinical aspects of EBV-associated lymphoid and epithelial malignancies and relates the association of each of these tumors with EBV infectivity. The genetic basis of tumorigenicity, including both EBV and cellular gene expression underlying cancer formation was also reviewed for these malignancies. All of the tumors mentioned in this review were found to be EBV-positive; however, some of them may also have EBV-negative correlation e.g. Hodgkin's disease and gastric carcinomas. 

Different epidemiological patterns were reported for these tumors depending on the geographical distribution, ethnic group of people, host age and gender...etc. Some of these tumors showed racial restrictions (e.g. NPC was known to be having special affinity to Asians as compared to other ethnic groups) while other co-factors like smoke, salted food intake and other kinds of food were also observed to be linked to some of these tumors as predisposing factors. Generally, different clinical presentations were reported for different tumors and this was governed by the tissues and sites affected as well as the complications that develop.

Generally, the various genetic mechanisms adopted by EBV to induce tumorigenesis of infected cells are complex, but orchestrated processes. These include expression of some viral proteins and shut down of others. The expression of the latent genes of the virus, namely LMP1, LMP2A, LMP2B, EBNA1 and EBNA2, was important with LMP1 reported as the principal oncogenic factor of EBV while LMP2 as a mediator of tumor cell survival. EBNA1 is the protein required to bind the EBV genome to host chromosomes and thus allow the transfer of the viral DNA to the progeny cells during cellular multiplication. EBNA2 is a transactivator of LMP1. These EBV-associated malignancies develop despite strong immune responses to the virus but the virus adopt several strategies for immune evasion including expression of latent genes during the latency stage. In conclusion, an improved understanding of the molecular mechanisms leading to the development of these malignancies, which has accumulated in recent years, may surely come up with better prognostic, treatment and management regimens for them in the future.

## Figures and Tables

**Table 1:** Expression of EBV-latency associated genes during latency programs [87].

Latency program	EBV gene expressed						occurrence
	EBERs	EBNA1	LMP1	LMP2A	EBNA2	EBNA3	
0	+	ND	-	+	-	-	Memory B cells in peripheral blood
I	+	+	-	-	-	-	BL, PEL
II	+	+	+	+	-	-	HD
III	+	+	+	+	+	+	PTLD
IV	ND	ND	-	ND	+	ND	Infectious mononucleosis, PTLD

BL, Burkitt lymphoma; HD, Hodgkin lymphoma; ND, Not determined; PEL, primary effusion lymphoma; PTLD, post-transplant lymphoproliferative disease; +, expressed; -, not expressed.

**Table 2:** The role of different EBV-encoded latent genes during latency and tumorigenesis [102].

Latent genes	Role of genes
*EBNA-1*	Transactivator of viral latent genes and host genes; responsible for episome replication, segregation and persistence of viral genome; involved in p53 degradation and oncogenesis.
*EBNA-LP*	Transcriptional co-activator of EBNA-2-dependent viral and cellular gene transcription; It is essential for EBV-mediated B-cell transformation.
*EBNA-2*	Activates viral and cellular gene transcription for transformation. It is critical for EBV-mediated B-cell transformation.
*EBNA-3A*	A co-activator of EBNA-2, downregulate cMyc transcription and block EBNA-2 activation effects; and induce CDKN2 and chemokines. It is essential for EBV-mediated B-cell transformation.
*EBNA-3B*	A co-activator of EBNA-2; dispensable for B-cell transformation; viral tumor suppressor; and up regulates CXCL10. EBNA-3B-knockout induces DLBCL-like tumors.
*EBNA-3C*	ates with EBNA-2 host * CXCR4* and *CXCL12* genes; overcomes EBV-infection-mediated DNA damage response; promotes cell proliferation; induces G1 arrests; It is essential for EBV-mediated B-cell transformation.
*LMP-1*	Mimics the constitutively active form of CD40, activates NF-κB, JNK and p38 pathways; is critical for EBV-mediated B-cell transformation.
*LMP-2A*	Responsible for constitutive activation of the ERK/MAPK pathway224; blocks antigen-dependent BCR signaling; induces B-cell lymphoma in transgenic condition. It is important but not essential for *in vitro* primary B-lymphocyte growth transformation.
*EBER*	Augments colony formation and induces growth; confers cells resistance to PKR-dependent apoptosis; induces cytokines and modulates innate immune response; contributes to EBV oncogenesis.
*miRNAs*	Has a role in sustaining latently infected cells. BHRF1 miRNA and BART miRNAs interfere with apoptosis.

BART, BamHI-A region rightward transcript; BHRF1, BamHI fragment H rightward open reading frame 1; EBV, Epstein-Barr virus; EBER, EBV-encoded nuclear antigen; EBNA, EBV nuclear antigen; LMP, Latent membrane protein, BHRF1, BamHI fragment H rightward open reading frame 1; MAPK, mitogen-activated protein kinase; NF-κB, nuclear factor-κB; BCR, B cell receptor; PKR, RNA-dependent protein kinase; JNK, c-Jun N-terminal kinase.
